# Why and how protein aggregation has to be studied *in vivo*

**DOI:** 10.1186/1475-2859-12-17

**Published:** 2013-02-15

**Authors:** Diletta Ami, Antonino Natalello, Marina Lotti, Silvia Maria Doglia

**Affiliations:** 1 Department of Biotechnology and Biosciences, University of Milano-Bicocca, Piazza della Scienza 2, 20126, Milano, Italy; 2 Department of Physics “G. Occhialini”, University of Milano-Bicocca, Piazza della Scienza 3, 20126, Milano, Italy

**Keywords:** Amyloids, Inclusion bodies, Intact cells, Protein aggregation, Spectroscopy

## Abstract

The understanding of protein aggregation is a central issue in different fields of protein science, from the heterologous protein production in biotechnology to amyloid aggregation in several neurodegenerative and systemic diseases. To this goal, it became more and more evident the crucial relevance of studying protein aggregation in the complex cellular environment, since it allows to take into account the cellular components affecting protein aggregation, such as chaperones, proteases, and molecular crowding. Here, we discuss the use of several biochemical and biophysical approaches that can be employed to monitor protein aggregation within intact cells, focusing in particular on bacteria that are widely employed as microbial cell factories.

## 

Protein aggregation is a relevant process in different fields of biomedicine and biotechnology. Indeed, many diseases are associated to the deposition of amyloid aggregates [1], while the formation of inclusion bodies (IBs) often occurs during the production of heterologous proteins [2,3]. In particular, bacterial IBs, for a long time considered a bottleneck during recombinant protein production, have recently gained attention [4,5] as a precious source of active recombinant proteins [6-8], as well as a model system for amyloid studies [9-15]. Moreover, the peculiar structural properties of IBs and the observation that the aggregated proteins can retain their activity opened the possibility to use IBs in bio-catalysis [16], regenerative medicine [17], and in the controlled delivery of therapeutic polypeptides [18,19].

Protein misfolding and aggregation have been extensively studied in the test tube, therefore under conditions that are far from the physiological and pathological ones. For this reason, in order to take into account the complexity of the cellular environment that plays a crucial role in tuning protein aggregation [20], it is important to extend these investigations to intact cells [21].

In this commentary we focalized our attention on the different approaches that allow to monitor protein aggregation within bacterial cells (Table 1). We should note that most of these approaches have been successfully applied to monitor protein aggregation also within intact eukaryotic cells, including yeasts and mammals.

**Table 1:** Methods for the study of protein aggregation in intact cells

**Approach**		**Measurement methods**	**Application examples**
❖ **Genetically encoded fusion tags**			
✓ **Fusion of the target polypeptide with a fluorescent protein or an enzyme**	Reduction of fluorescence or of enzymatic activity after aggregation; detection of functional polypeptides within active IBs	Bulk cell fluorescence; fluorescence microscopy; flow cytometry; enzymatic activity	Monitoring of protein aggregation within intact cells [31]; localization of functional polypeptides within IBs [22]; formation of active IBs [16,23,24]; screening of aggregation inhibitors [26]
✓ **Fusion of the target polypeptide with the tetra-Cys tag**	Formation of hyperfluorescent aggregates in presence of FlAsH		
❖ **Conformational sensitive dyes**			
✓ **Thioflavin-S**	Th-S fluorescence reports on amyloid-like structure of the protein aggregates	Bulk cell fluorescence; fluorescence microscopy; flow cytometry	Detection of amyloid-like aggregates within intact cells [32]
❖ **Direct spectroscopic detection of structural properties**			
✓ **FTIR**	Monitoring of intermolecular β-sheet structures in IBs	Label-free intact cell (micro)spectroscopy	Monitoring of protein aggregation whithin intact cells [35,39]
✓ **NMR**	Detailed structural information of the protein embedded within IBs	Solid-state NMR of whole cells	Detection of native-like structures [43]
❖ **Aggregation sensitive reporters**			
✓ **Reporter protein under an aggregation sensitive promoter**	Protein aggregation induces the expression of the reporter protein. The measured fluorescence or enzymatic activity of the reporter protein is related to the level of aggregation within cells	Enzymatic activity; fluorescence	Monitoring of protein aggregation within intact cells [37,45]

Among the most employed methods to study protein aggregation *in situ*, some are based on the fluorescence detection of genetically encoded fusion tags, or of conformational-sensitive fluorescent dyes. In the first case, one of the most important tools is represented by the green fluorescent protein (GFP) and its variants, such as the yellow, the blue and the red, used to obtain fluorescent chimera-proteins, easily detectable by fluorescence microscopy and flow-cytometry.

This approach has been applied, for instance, to investigate the presence of functional proteins embedded in bacterial IBs [22-24]. Interestingly, in recent works it has been observed that the fusion of self-assembling or surfactant-like peptides to different proteins makes it possible to obtain active IBs, whose formation was detected *in vivo* monitoring the fluorescence of GFP - taken as a model system - fused to the peptide. Indeed, the bacterial cell images, obtained by confocal microscopy, showed a diffuse fluorescence when GFP was expressed alone, in a soluble form. When, instead, the GFP was expressed fused to the self-assembling or surfactant-like peptide, the fluorescence appeared localized in the cell, indicating the formation of active IBs [23,24]. Moreover the use of GFP tag as a reporter for corrected folding has been employed for the screening of Aβ mutations and chemical compounds able to tune the aggregation propensity of the peptide. In particular, it should be noted that the fluorescence of the fusion protein in intact cells was found to be inversely correlated with the aggregation of the Aβ-GFP fusion protein [25-27].

Noteworthy, the fusion with fluorescent proteins has been also employed to investigate the mechanism of protein deposition at the single cell level [28] and the specificity of protein-protein interaction during *in vivo* protein deposition. To this aim, for instance, Morell and colleagues performed Förster resonance energy transfer (FRET) experiments in prokaryotic cells, labeling two self-aggregating proteins, the Aβ42 amyloid peptide and the VP1 capsid protein, with proper fluorescent protein variants [29]. In this way, the specificity of protein deposition was indicated by a higher FRET efficiency, observed when the two dyes were fused to the same poly peptide, rather than to the different ones.

Other applications based on fluorescence analysis to detect, in real time, protein aggregation *in vivo* include the labeling of the target protein with a tetra-cysteine sequence (Cys-Cys-X-Y-Cys-Cys), which specifically binds the bis-arsenical fluorescein-based dye (FIAsH) [30]. This smart approach enables to monitor the formation of hyperfluorescent aggregates within intact cells, by simply detecting the bulk cell fluorescence or by fluorescence microscopy [30,31].

Protein aggregation can be also studied *in vivo* using conformational-sensitive dyes, such as the thioflavin-S (Th-S), whose fluorescence spectroscopic features change upon interaction with amyloid aggregates. As recently reported in the literature, the capability of Th-S to be internalized in bacterial cells has been exploited to detect intracellular amyloid-like aggregates by fluorescence spectroscopy, microscopy and flow cytometry. Interestingly, this approach can represent a new tool to screen the effects of amyloid inhibitors in an intracellular environment [32].

Among the spectroscopic techniques that allow to study protein aggregation in intact cells, Fourier transform infrared (FTIR) spectroscopy offers the advantage to be a label-free tool. In particular, the detection of protein aggregates is based on the presence of a specific marker band due to the formation of intermolecular β-sheet structures [33,34]. Following this approach, it has been possible to monitor the kinetics of IB formation within growing *E. coli* cells, under different expression conditions [35]. Interestingly, since the infrared response of an intact cell represents a chemical fingerprint of its main biomolecules [36], the IR spectral analysis makes it possible to obtain also complementary information on cell processes that accompany protein aggregation, including for instance the effects on cell membranes [37].

Moreover, the IR study of extracted IBs allows to obtain important information on the structural properties of the aggregated protein [34,38,39], and in particular to detect the presence of native-like secondary structures of the proteins within IBs. For these reasons, the IR approach is a useful tool to identify the best conditions that enable to modulate not only the level of protein aggregation, but also the quality of the protein inside the IBs.

A more detailed structural information of the protein embedded within IBs can be obtained by nuclear magnetic resonance (NMR) spectroscopy, a technique that was applied not only to characterize isolated [40-42] IBs, but also IBs within cells [43]. For instance, in the pioneering work of Curtis-Fiske and colleagues, solid state NMR was applied to study whole bacterial cells expressing the HA2 subunit of the influenza virus hemagglutinin protein in form of IBs. In this way, labeling the backbone carbonyl and nitrogen (^13^CO and ^15^N) for each amino-acid, it has been possible to identify the localization of native-like α-helices of the protein functional domain, and to reveal also the protein conformational heterogeneity within IBs [43].

Finally, the evaluation of protein aggregation within intact cells could be also tackled by a biochemical approach based on the use of gene promoters specifically triggered by protein misfolding and aggregation [44-46]. For instance, the expression of the β-galactosidase reporter under the control of the chaperone IbpB promoter, specifically activated by misfolded proteins, allowed the estimation of protein aggregation accumulated inside the cell [45]. By this approach, together with complementary biochemical and biophysical analyses, it has been studied the recombinant expression of the glutathione-S-transferase and its fusion with GFP, whose aggregation can be tuned by changing the expression conditions. Interestingly, it has been found that in this model system misfolded proteins and soluble aggregates - but not the soluble native protein nor IBs - lead to a significant reorganization of the cell membranes and of the host protein expression [37], a relevant result in the proteotoxicity context.

## Conclusions

We underline here the need to extend the study of protein aggregation in an intracellular environment in the presence of factors - such as chaperones, proteases, and the molecular crowding - that can affect in a crucial way the aggregation process *in vivo*.

Indeed, it will be necessary to complement studies in the test tube with those in intact cells, not only to reach a better comprehension of the mechanisms underlying protein aggregation, but also to identify the factors that can modulate aggregation, such as protein expression conditions, mutations, and the effects of chemical compounds.

In this view, it will be highly desirable to further develop methods that might enable investigations in intact cells, not only for the basic understanding of aggregation *in situ*, but also for applications in recombinant protein productions and for the screening of compounds inhibiting aggregation, a relevant issue in medical therapies.

## Abbreviations

FIAsH: Bis-arsenical fluorescein-based dye; FRET: Förster resonance energy transfer; FTIR: Fourier transform infrared; GFP: Green fluorescent protein; IBs: Inclusion bodies; NMR: Nuclear magnetic resonance; Th-S: Thioflavin-S.

## Competing interests

The authors declare that they have no competing interests.
